# The Roles of Rods, Cones, and Melanopsin in Photoresponses of M4 Intrinsically Photosensitive Retinal Ganglion Cells (ipRGCs) and Optokinetic Visual Behavior

**DOI:** 10.3389/fncel.2018.00203

**Published:** 2018-07-12

**Authors:** Melanie M. Schroeder, Krystal R. Harrison, Elizabeth R. Jaeckel, Hunter N. Berger, Xiwu Zhao, Michael P. Flannery, Emma C. St. Pierre, Nancy Pateqi, Agnieszka Jachimska, Andrew P. Chervenak, Kwoon Y. Wong

**Affiliations:** ^1^Department of Ophthalmology & Visual Sciences, University of Michigan, Ann Arbor, MI, United States; ^2^Department of Molecular, Cellular & Developmental Biology, University of Michigan, Ann Arbor, MI, United States

**Keywords:** retina, intrinsically photosensitive retinal ganglion cell (ipRGC), melanopsin, rod, cone, photoreceptor, vision, visual behavior

## Abstract

Intrinsically photosensitive retinal ganglion cells (ipRGCs) mediate not only image-forming vision like other ganglion cells, but also non-image-forming physiological responses to light such as pupil constriction and circadian photoentrainment. All ipRGCs respond to light through their endogenous photopigment melanopsin as well as rod/cone-driven synaptic inputs. A major knowledge gap is how melanopsin, rods, and cones differentially drive ipRGC photoresponses and image-forming vision. We whole-cell-recorded from M4-type ipRGCs lacking melanopsin, rod input, or cone input to dissect the roles of each component in ipRGCs' responses to steady and temporally modulated (≥0.3 Hz) lights. We also used a behavioral assay to determine how the elimination of melanopsin, rod, or cone function impacts the optokinetic visual behavior of mice. Results showed that the initial, transient peak in an M4 cell's responses to 10-s light steps arises from rod and cone inputs. Both the sustainability and poststimulus persistence of these light-step responses depend only on rod and/or cone inputs, which is unexpected because these ipRGC photoresponse properties have often been attributed primarily to melanopsin. For temporally varying stimuli, the enhancement of response sustainedness involves melanopsin, whereas stimulus tracking is mediated by rod and cone inputs. Finally, the behavioral assay showed that while all three photoreceptive systems are nearly equally important for contrast sensitivity, only cones and rods contribute to spatial acuity.

## Introduction

Intrinsically photosensitive retinal ganglion cells (ipRGCs) use the photopigment melanopsin (gene symbol *Opn4*) and an invertebrate-like phototransduction cascade to generate excitatory responses to light (Provencio et al., 1998; Berson et al., 2002; Hattar et al., 2002; Graham et al., 2008; Xue et al., 2011). In addition to their intrinsic photosensitivity, all ipRGCs exhibit rod- and cone-mediated, predominantly depolarizing light responses (Dacey et al., 2005; Wong et al., 2007; Schmidt and Kofuji, 2010, 2011; Weng et al., 2013; Zhao et al., 2014). A functional role of ipRGCs appears to be the measurement of ambient light levels, as the amplitude of their very tonic photoresponses faithfully tracks absolute light intensity, i.e., irradiance (Dacey et al., 2005; Wong, 2012). Though ipRGCs were originally thought to drive primarily non-image-forming visual behaviors such as pupil constriction, suppression of locomotion in nocturnal rodents (“negative masking”), and circadian photoentrainment (Berson, 2003; Hattar et al., 2006), they are now known to also innervate image-forming visual centers and mediate pattern vision, brightness discrimination, and contrast detection (Ecker et al., 2010; Brown et al., 2012; Schmidt et al., 2014).

Because ipRGCs receive rod/cone input like all other ganglion cells, a frequently asked question is why they need melanopsin. In other words, is melanopsin necessary for any aspect of an ipRGC's light response? The extraordinary sustainedness of melanopsin-based photoresponses (Berson et al., 2002; Melyan et al., 2005; Panda et al., 2005; Qiu et al., 2005) and the observation that all ipRGCs display far more sustained light responses than conventional, melanopsin-less ganglion cells (Wong et al., 2007) led to the notion that melanopsin is necessary for ipRGCs to respond tonically to light, but more recent work showed that the rod/cone input alone can evoke similarly sustained light responses (Wong, 2012; Zhao et al., 2017). Another aspect of the ipRGC photoresponse that was initially thought to require melanopsin was the response's poststimulus persistence, as early recordings found rod/cone-driven ipRGC photoresponses to end abruptly at stimulus offset (Dacey et al., 2005; Wong et al., 2007). One subsequent study indeed found a dramatic reduction of the poststimulus persistence when melanopsin was knocked out (Schmidt et al., 2014), but another paper reported that melanopsin-knockout ipRGCs still gave photoresponses persisting for minutes after stimulus offset (Wong, 2012). Thus, the roles of melanopsin in the ipRGC photoresponse remain unclear.

The roles of rod/cone inputs are better understood, e.g., they accelerate an ipRGC's light response, lower the intensity threshold (Dacey et al., 2005; Wong et al., 2007), and create several receptive field properties including center/surround antagonism (Estevez et al., 2012; Zhao et al., 2014), speed tuning (Zhao et al., 2014), and color opponency (Dacey et al., 2005; Stabio et al., 2017). However, how rod input and cone input differentially contribute to an ipRGC's photoresponse is largely unknown, aside from their ~2 log sensitivity difference (Zhao et al., 2014).

Here, we help fill these knowledge gaps by examining how selectively disrupting melanopsin, rod, or cone function affects ipRGCs' responses to steady and temporally modulated light stimuli, to test the hypothesis that each photoreceptive component is necessary for certain aspects of these responses such as sustainedness, poststimulus persistence, and temporal tracking. There are at least five morphological types of rodent ipRGCs: M1 cells have sparse dendrites terminating exclusively in the OFF sublamina of the inner plexiform layer; M2 cells have sparse dendrites stratifying exclusively in the ON sublamina; M3 cells stratify in both sublaminas; M4 cells have very large somas and moderately dense, ON-stratifying dendrites that branch in a radiate pattern; and M5 cells have very dense, ON-stratifying dendrites with a bushy appearance and relatively small field size (Viney et al., 2007; Ecker et al., 2010; Reifler et al., 2015b). These ipRGC types are physiologically diverse (Ecker et al., 2010; Schmidt and Kofuji, 2010, 2011; Hu et al., 2013; Zhao et al., 2014). We chose to study the M4 type as its distinctively large soma allows it to be identified easily (Pack et al., 2015), and because a highly relevant prior study also focused on M4 (Schmidt et al., 2014). Since M4 ipRGCs participate in image-forming vision (Estevez et al., 2012; Schmidt et al., 2014), we also investigated the roles of the three photoreceptive systems in spatial visual behavior by studying how selectively disrupting each system affects optokinetic tracking behavior.

## Materials and methods

### Animals

All procedures were approved by the Institutional Animal Care and Use Committee at the University of Michigan. Four mouse strains were used: (1) *Opn4*^*Cre*/*Cre*^ mice (“melanopsin-knockout mice”) in which both copies of the melanopsin open reading frame are replaced by the gene encoding Cre recombinase (Ecker et al., 2010); (2) *Gnat1*^−/−^ mice with non-photosensitive rods (Calvert et al., 2000); (3) *Gnat2*^*cpfl*3^ mice with non-photosensitive cones (Chang et al., 2006); and (4) B6129SF2/J wild-type mice made by crossing C57BL/6J with 129S1/SvImJ mice (Jackson Laboratory stock # 101045). Animals were 4–8 months old, included both sexes, and were housed in a 12 h light 12 h dark cycle with all experiments done during the light phase.

### Whole-cell recording of M4-type ipRGCs

A mouse was dark-adapted overnight before the day of an experiment. Under dim red light, the mouse was euthanized using CO_2_ followed by cervical dislocation, and its eyes were enucleated and put in Ames' medium gassed with 95% O_2_ and 5% CO_2_. Under infrared-based night vision viewers, the retinas were isolated from the retinal pigment epithelium (RPE), the vitreous was removed using forceps, and each retina was cut into quadrants. A quadrant was flattened on the bottom of a superfusion chamber with the ganglion cell side up and superfused by bubbled Ames' medium at ~3 mL min^−1^, with temperature maintained at 33°C. The ganglion cell layer was viewed under infrared transillumination, and whole-cell recordings were made from the largest somas using an internal solution containing (in mM) 120 K-gluconate; 5 NaCl; 4 KCl; 10 HEPES; 2 EGTA; 4 Mg-ATP; 0.3 Na-GTP; 7 Tris-phosphocreatine; ~0.1% Lucifer Yellow for visualizing cellular morphologies; and KOH to set pH at 7.3.

**Figure 1:**
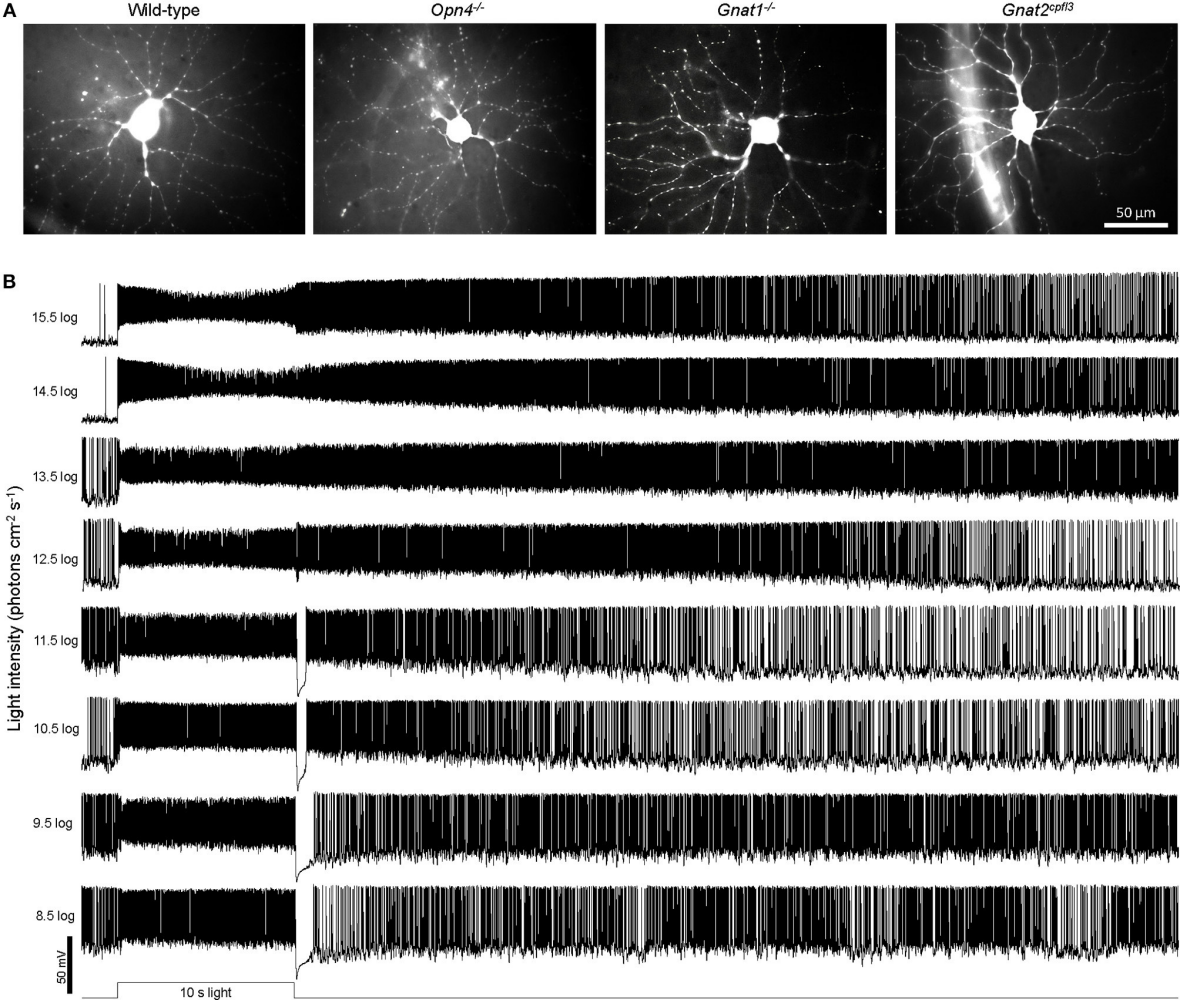
Examples of cellular morphologies and light-step responses. **(A)** Representative Lucifer Yellow fills of M4 ipRGCs from the four genotypes studied: wild-type, *Opn4*^−/−^, *Gnat1*^−/−^ (which lacks rod photosensitivity), and *Gnat2*^*cpfl*3^ (which lacks cone photosensitivity). **(B)** Representative light-step responses recorded from a wild-type M4 ipRGC.

The retina was kept in darkness except when presented with test stimuli, which were all full-field light. In the experiment measuring responses to a sum-of-sinusoids stimulus lasting 23.0 s, the temporal pattern of light intensity modulation was generated by summing 21 equal-amplitude sinusoids of different frequencies (in Hz): 0.3, 0.4, 0.5, 0.7, 0.95, 1.3, 1.9, 2.45, 3.1, 4.1, 5.3, 6.3, 7.2, 8.9, 10.7, 13.9, 16.7, 19.6, 23.1, 27.5, and 31.7. This stimulus was identical to the “high contrast” sum-of-sinusoids stimulus described in (Howlett et al., 2017) with the highest-intensity point being ~3.4 log units above the lowest-intensity point (Figure 8A), except that: (1) our light source was a 470 nm light-emitting diode rather than white light; and (2) the intensity range was 9.1 log−12.5 log photons cm^−2^ s^−1^, with a mean intensity of 10.8 log photons cm^−2^ s^−1^ which is in the high mesopic range (Dacey et al., 2005). Light intensity was varied by a microcontroller board (Arduino; Scarmagno, Italy) using pulse width modulation, and the light was delivered to the retina via the objective lens. In all other experiments, the stimuli were 480 nm light presented from below the transparent bottom of the superfusion chamber. Stimulus presentation orders and interstimulus intervals were standardized across all cells. Each cell was presented with just one of the three sets of stimuli, i.e., light steps, flickers, or the sum-of-sinusoids stimulus. Each cell's identity as an M4 ipRGC was verified first by its tonic depolarizing response lasting throughout a 10 s light step (10.5 log−11.5 log photons cm^−2^ s^−1^) and then by post-recording visualization of its morphology (Ecker et al., 2010; Estevez et al., 2012). All cells with other light-step responses and/or morphologies were discarded. M4 cells in all genotypes were morphologically similar (Figure 1A), confirming that the lack of rod, cone, or melanopsin activity did not significantly alter their cellular morphologies. To produce the photos in Figure 1A, each cell's dye fill was imaged at 3–8 focal planes using epifluorescence microscopy. After all out-of-focus cellular structures in each image had been masked manually, all focal planes were z-projected using ImageJ software (National Institutes of Health, Bethesda MD).

### Behavioral assay

All experiments employed the virtual optokinetic system (Prusky et al., 2004) sold by CerebralMechanics (Lethbride AB, Canada). Briefly, a mouse was placed on a platform at the center of an arena formed by four computer monitors, and the lid of the apparatus was closed prior to testing. In each trial, the monitors presented a grayscale vertical sine wave grating that drifted either clockwise or counterclockwise at 12 deg/s, and the mouse's tracking behavior was assessed by an experimenter blind to the animal's genotype. Tracking was determined by identifying tracking episodes lasting 1–1.5 s. The two parameters we tested were spatial frequency, and Michelson contrast defined as (max luminance – min luminance) / (max luminance + min luminance). Contrast sensitivity was the reciprocal of the Michelson contrast threshold. For each mouse, acuity testing lasted 7–15 min while contrast sensitivity testing lasted 15–30 min. Light intensity at the mouse's cornea was about 12 μW cm^−2^, corresponding very approximately to 13.5 log photons cm^−2^ s^−1^. When measuring this intensity, wavelengths exceeding 700 nm were blocked using a low-pass filter as they are irrelevant to mouse retinal photoreception.

### Data analysis

For analyzing graded membrane potentials (*V*_m_), action potentials in the whole-cell recordings were first filtered out using a 20 Hz low-pass filter (for the light-step and flicker responses) or a 40 Hz low-pass filter (for the 21-frequency responses). Six parameters were analyzed:

1. *Peak amplitude of a light-step response*:
  $$\begin{array}{l} \left(\text{most positive }{\text{V}}_{\text{m}}\text{ during the first }1\mathrm{s of the light step}\right)\\ \;-\left(\text{prestimulus }{\text{V}}_{\text{m}}\right) \end{array}$$
2. *Final-to-peak amplitude ratio of a light-step response*:
  $$\begin{array}{l} \frac{\left(\text{mean }\text{Vm during the final }500\text{ ms of the light step}\right)\;-\;\left(\text{prestimulus Vm}\right)}{\left(\text{most positive }\text{Vm during the first }1\text{ s of the light step}\right)\;-\;\left(\text{prestimulus Vm}\right)} \end{array}$$
3. *Poststimulus persistence of a light-step response*:
  $$\begin{array}{l} \left(\text{mean }{\text{V}}_{\text{m}}\;10\text{s to }50\mathrm{s after stimulus offset}\right)\\ \;-\left(\text{prestimulus }{\text{V}}_{\text{m}}\right) \end{array}$$
4. *Amplitude of the response to the final pulse in a flicker*:
  $$\begin{array}{l} \left(\text{most positive }{\text{V}}_{\text{m}}\text{ during the final pulse response}\right)\\ \;-\left(\text{most negative }{\text{V}}_{\text{m}}\text{ just before the final pulse response}\right) \end{array}$$
5. *Final-to-peak amplitude ratio of a flicker response*:
  $$\begin{array}{l} \frac{\left(\text{most positive Vm during the final pulse response}\right)\;-\;\left(\text{prestimulus Vm}\right)}{\left(\text{most positive Vm during the first pulse response}\right)\;-\;\left(\text{prestimulus Vm}\right)} \end{array}$$
6. *Amplitudes in a sum-of-sinusoids response*: Fast Fourier transform was performed to calculate response amplitudes at the 21 frequencies present in the sum-of-sinusoids stimulus (Howlett et al., 2017).

When constructing spike histograms, action potentials in the unfiltered whole-cell recordings were counted and grouped into 1-s bins, with spike counts during the 5-s prestimulus baseline normalized to 0. To quantify the persistence of light-step responses, spike counts relative to the prestimulus baseline were summed 10–50 s after stimulus offset.

Statistical comparisons of the four genotypes were made using one-way ANOVA followed by post hoc Tukey tests. *P*-values smaller than 0.05 indicate significant differences.

## Results

### Responses to light steps

**Figure 2:**
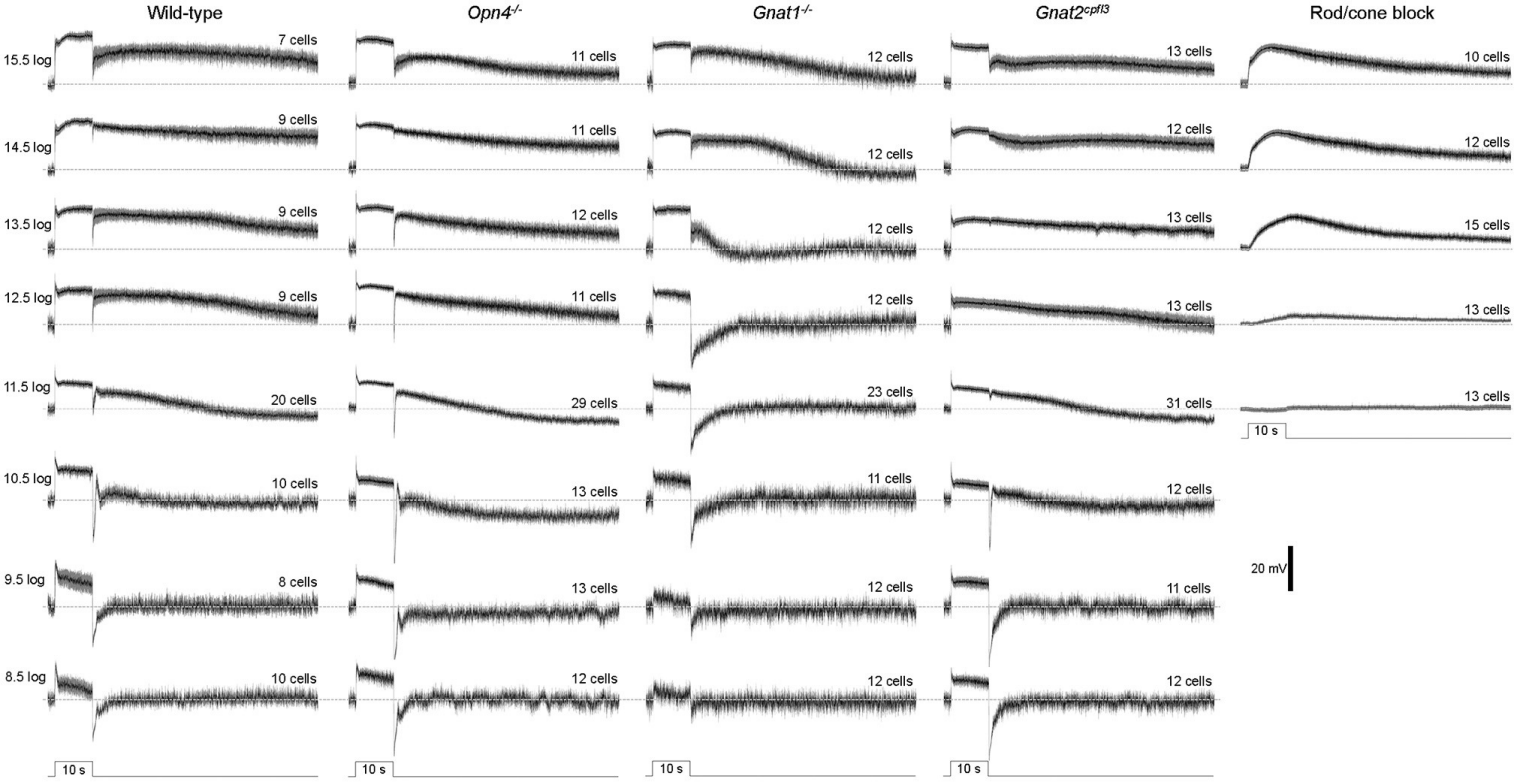
Light-step responses averaged from all cells tested. The black traces represent the mean values, and the surrounding gray areas denote S.E.M. The horizontal dashed lines mark the prestimulus baselines. *N* values are stated above each trace. The cells recorded during rod/cone signaling block included wild-type, *Gnat1*^−/−^ and *Gnat2*^*cpfl*3^ cells.

In the first whole-cell recording experiment, we presented each cell with an intensity series of 10-s light steps ranging from 8.5 log to 15.5 log photons cm^−2^ s^−1^. As exemplified by the representative responses in Figure 1B, M4 cells give excitatory spiking responses lasting the duration of the light steps, and at sufficiently high stimulus intensities, the excitation persists for many seconds after a transient hyperpolarization at light offset. To assess how melanopsin, rod input, and cone input contribute to these light-step responses, we tested wild-type, melanopsin-knockout, rod-functionless (*Gnat1*^−/−^), and cone-functionless (*Gnat2*^*cpfl*3^) M4 cells, and population averages of the recordings are shown in Figure 2. Even the lowest intensity of 8.5 log photons cm^−2^ s^−1^ evoked a clear response in the rod-functionless cells, suggesting it is above the cone threshold. To estimate the melanopsin activation threshold, we recorded from wild-type, *Gnat1*^−/−^ and *Gnat2*^*cpfl*3^ M4 cells in the presence of the rod/cone signaling blockers L-(+)-2-amino-4-phosphonobutyric acid (50 μM), 6,7-dinitroquinoxaline-2,3-dione (40 μM), and D-(-)-2-amino-5-phosphonopentanoic acid (25 μM) (Slaughter and Miller, 1981; Hensley et al., 1993), and found 12.5 log photons cm^−2^ s^−1^ to be the lowest intensity needed to stimulate melanopsin (Figure 2 *rightmost column*), the slight hyperpolarizing response at 11.5 log photons cm^−2^ s^−1^ was previously shown to be likely a photoelectric artifact (Zhao et al., 2014).

**Figure 3:**
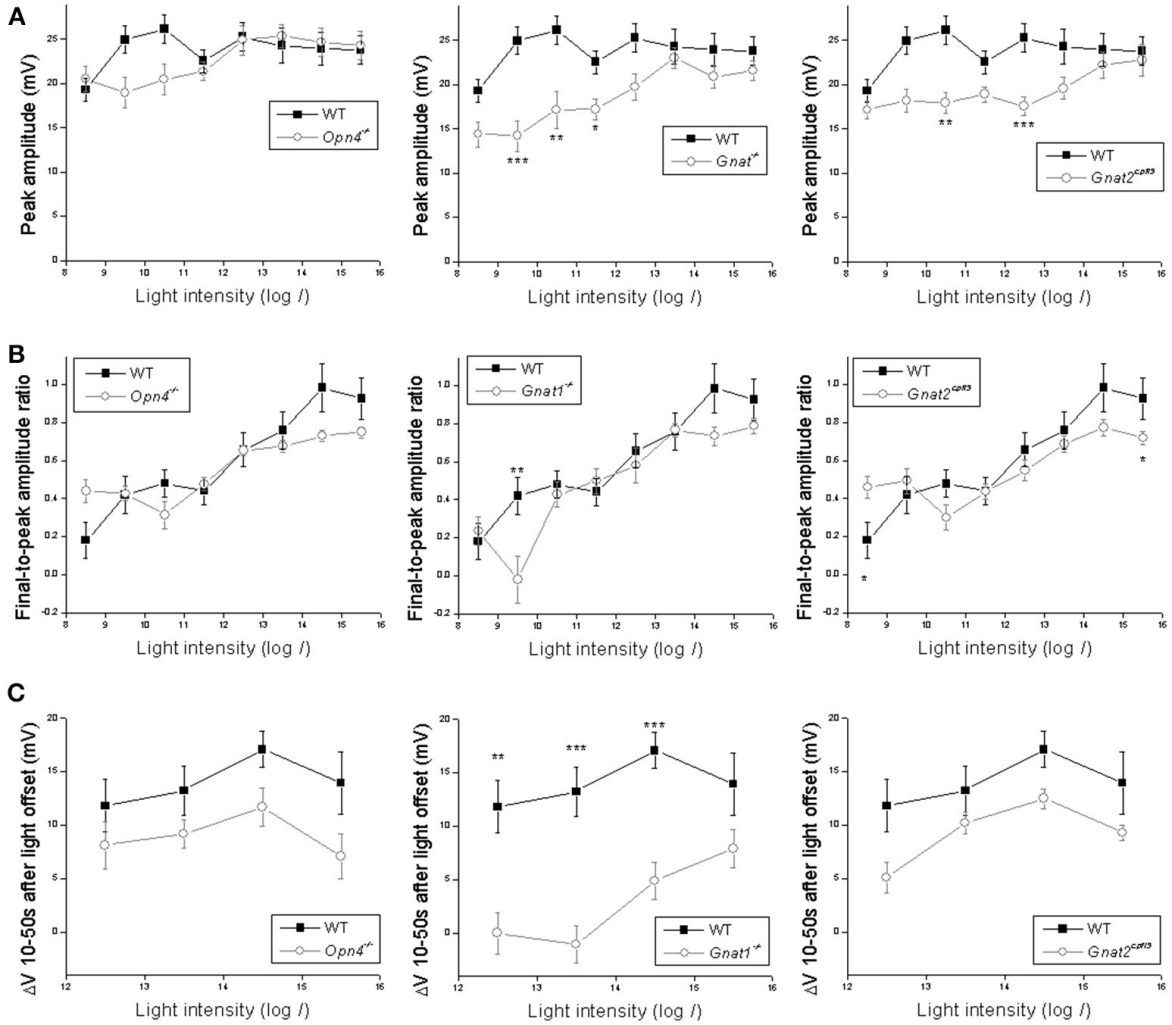
Analysis of the light-step responses. Comparisons are made between wild-type cells (*black squares*) and cells of the other genotypes (*open circles*) in three aspects of their light-step responses: **(A)** the peak response amplitude measured near light onset; **(B)** the ratio of the final response amplitude to the peak response amplitude, as a measure of response sustainedness; and **(C)** the mean *V*_m_ 10–50 s after light offset relative to the baseline, as a measure of the persistence of the light response. *N* values are identical to those shown in Figure 2. Error bars represent S.E.M. ^*^p < 0.05. ^**^p < 0.01. ^***^p < 0.001.

Statistical comparisons between the wild type's and the other genotypes' light-step responses are shown in Figure 3. The wild-type cells' peak response amplitudes (measured near light onset) were nearly “all-or-none,” with the lowest intensity inducing only slightly smaller responses than the higher intensities (Figure 3A). This could be due to Ca^2+^ spikes at the beginning of the responses (Hu et al., 2013) which were not eliminated by the 20 Hz low-pass filter. Knocking out melanopsin did not significantly affect any of the peak responses (Figure 3A *left*), whereas abolishing rod or cone function significantly reduced the peak response amplitude at several light intensities (Figure 3A *middle* and *right*).

In the second analysis, we calculated final-to-peak amplitude ratios to quantify the sustainability of the light-step responses—the more sustained a response is, the higher its final-to-peak ratio would be. Eliminating melanopsin did not significantly alter the final-to-peak ratio for any of the responses (Figure 3B *left*), while disrupting rod or cone input significantly altered this ratio at one and two intensities, respectively (Figure 3B *middle* and *right*).

In the third analysis, we quantified the poststimulus persistence of the light-step responses by averaging *V*_m_ relative to the baseline 10–50 s after light offset; we excluded the first 10 poststimulus seconds from this analysis to avoid the transient hyperpolarization seen in many responses. The wild-type cells had large persisting depolarizations at the four highest light intensities (Figure 1B). Abolishing rod input dramatically attenuated these poststimulus depolarizations at three of the four intensities (Figure 3C *middle*), but neither the melanopsin-knockout cells (Figure 3C *left*) nor the cone-functionless cells (Figure 3C *right*) had significantly reduced poststimulus depolarizations.

**Figure 4:**
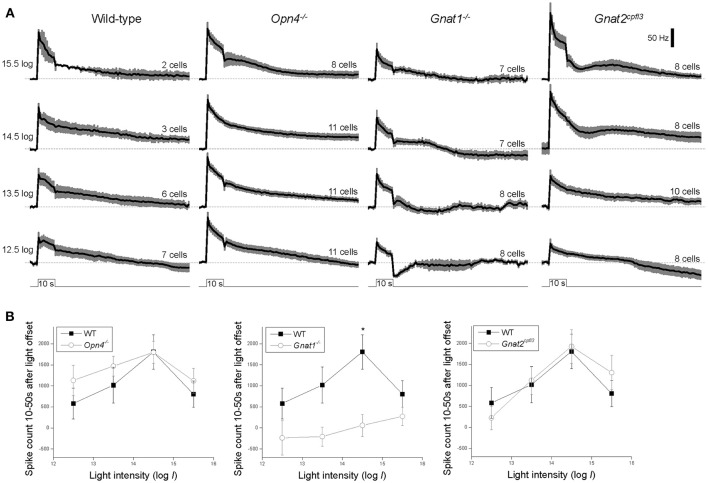
Spiking responses to the four highest-intensity light steps. **(A)** Spike histograms averaged from all cells tested, with 1-s bin width and baseline spiking normalized to 0. The black traces represent the means and the error bars denote S.E.M. *N* values are indicated above each histogram. **(B)** The number of light-evoked spikes recorded 10–50 s after stimulus offset, computed by summing the corresponding columns in the baseline-zeroed histograms shown in **(A)**. ^*^p < 0.05.

Contrary to our finding, a previous study reported a near-abolition of the poststimulus spiking increase in melanopsin-knockout M4 cells (Schmidt et al., 2014). Thus, we repeated the Figure 3C analysis by counting spikes instead of measuring *V*_m_. Figure 4A shows averaged spike histograms of the four genotypes' light-step responses at the four highest intensities, with prestimulus spiking normalized to zero, and Figure 4B plots the spike counts measured 10–50 s after light offset. This spike analysis confirmed that significant reduction in the poststimulus persistence was observed only in the M4 cells lacking rod input (Figure 4B *middle*), not in those without melanopsin (Figure 4B *left*) or cone input (Figure 4B *right*).

### Responses to temporally modulated light stimuli

**Figure 5:**
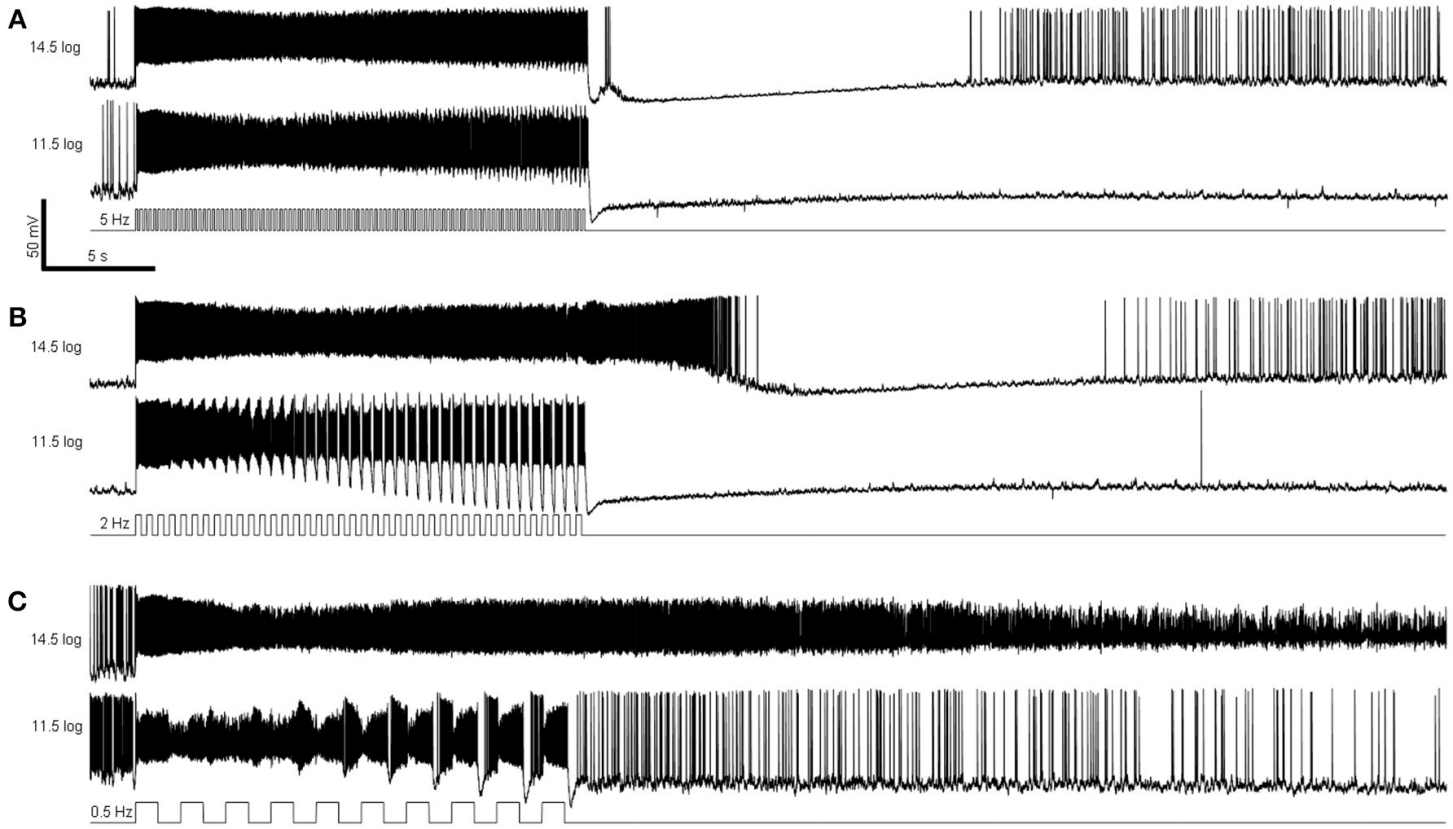
Examples of flicker responses. **(A)** Responses to a 5 Hz flicker. **(B)** Responses to a 2 Hz flicker. **(C)** Responses to a 0.5 Hz flicker. These responses were recorded from a wild-type M4 ipRGC.

**Figure 6:**
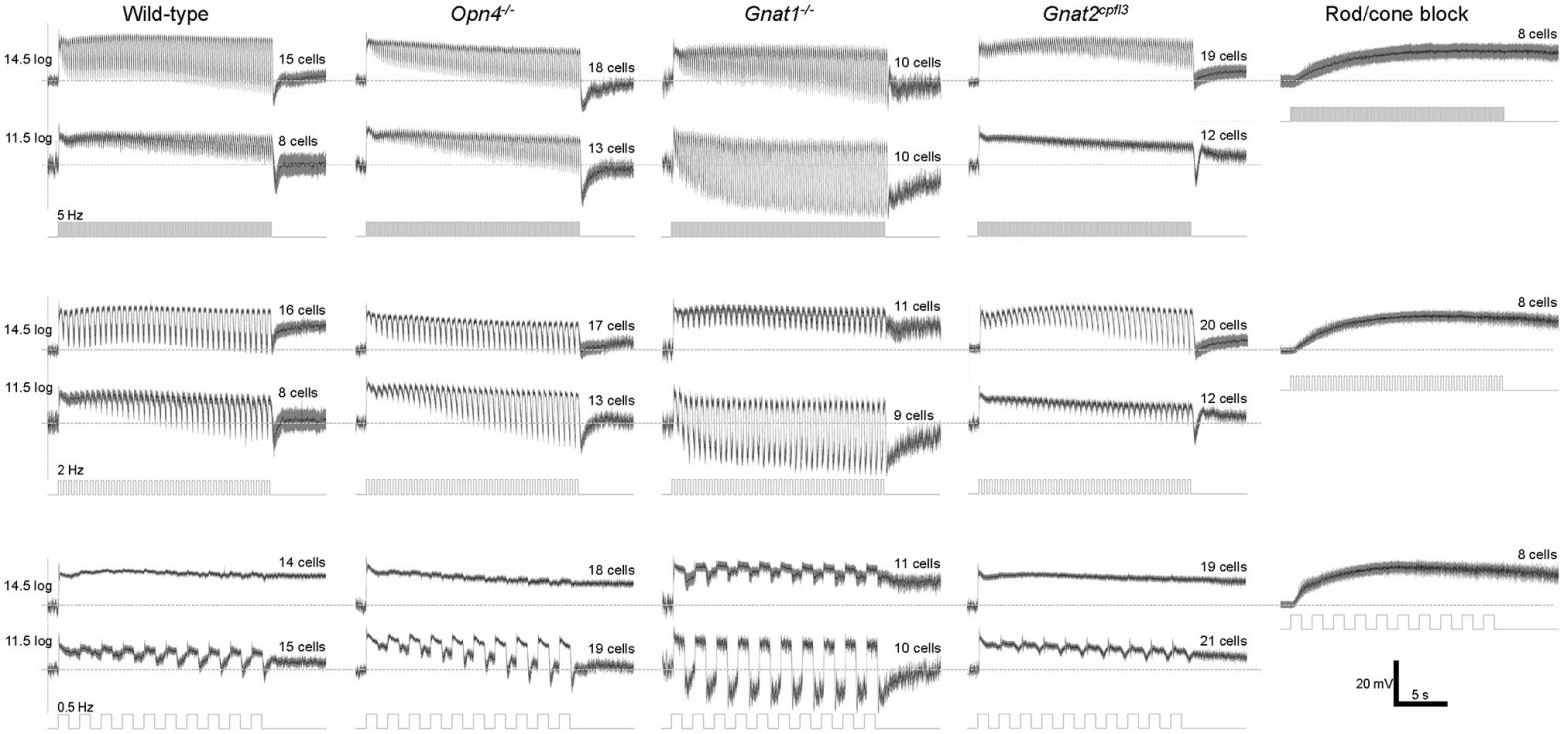
Flicker responses averaged from all cells tested. *N* values are indicated above each trace. The recordings made during rod/cone signaling block were obtained from wild-type, *Gnat1*^−/−^ and *Gnat2*^*cpfl*3^ cells.

The remaining whole-cell recording experiments assessed M4 cells' responses to temporally varying light. In the first experiment we presented 20-s flickers containing square light pulses that alternated with darkness, at 3 frequencies (0.5, 2, and 5 Hz) and 2 intensities (11.5 log and 14.5 log photons cm^−2^ s^−1^). Representative recordings are shown in Figure 5, and population averages in Figure 6. In the presence of normal Ames' medium, cells of the four genotypes seemed to show varying abilities to track the individual light pulses. Responses to the individual pulses gradually increased over the course of most flickers, probably indicating adaptation (Figure 6 first four *columns*). By contrast, melanopsin-only responses recorded during rod/cone block did not show any tracking of the pulses (Figure 6 *rightmost column*), indicating that synaptic input is necessary for pulse tracking.

**Figure 7:**
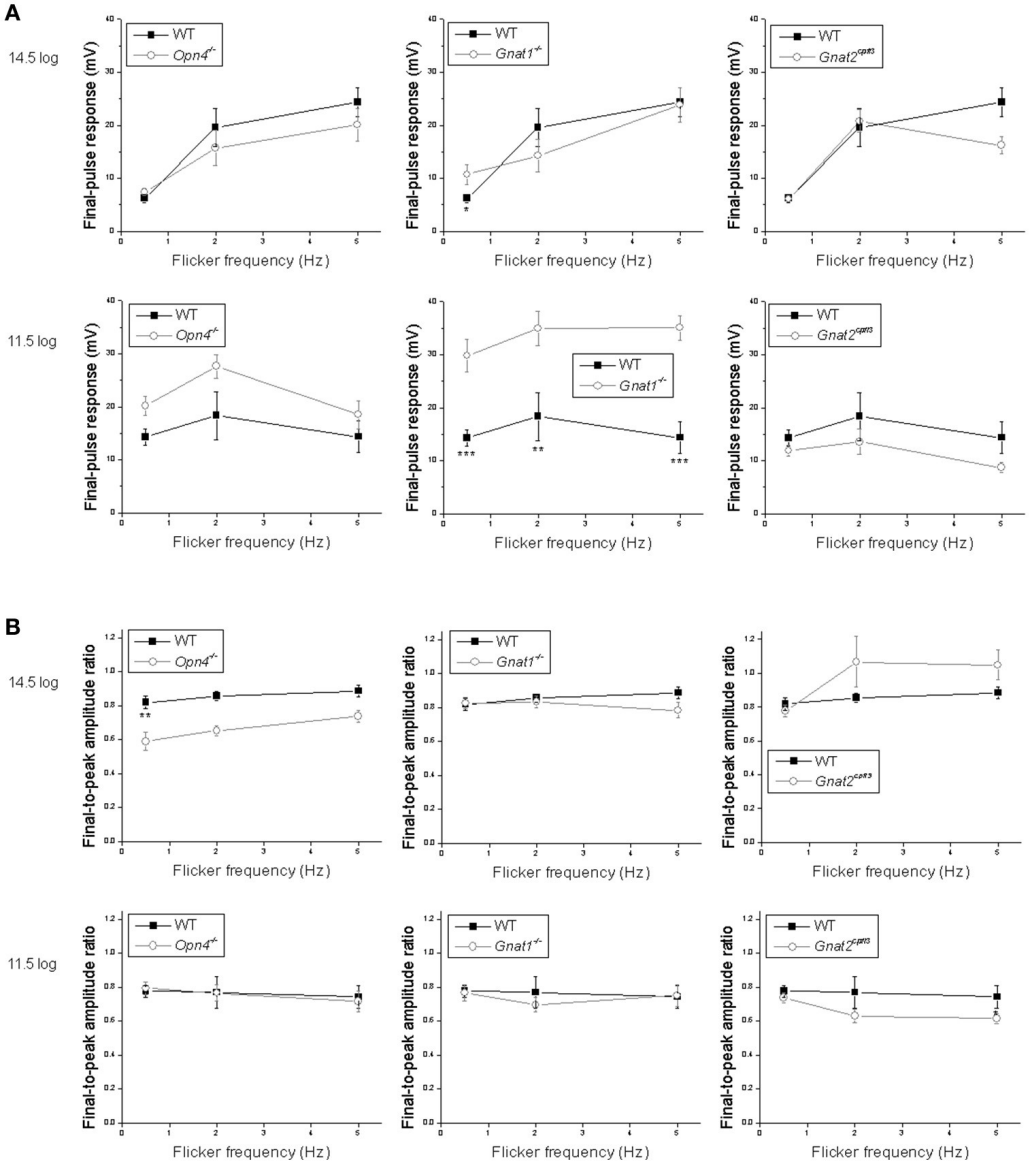
Analysis of the flicker responses. Comparisons are made between wild-type cells (*black squares*) and the other genotypes (*open circles*) in two aspects of their flicker responses: **(A)** the peak amplitude of the response to the final pulse in the flicker, measured relative to the trough preceding this response; and **(B)** the ratio of the peak of the final-pulse response to the peak of the first-pulse response, as a measure of the sustainedness of the entire flicker response. *N* values are identical to those shown in Figure 6. ^*^p < 0.05, ^**^p < 0.01, ^***^p < 0.001.

Statistical comparisons between the wild type's and the other genotypes' flicker responses are shown in Figure 7. To quantify a cell's ability to track the light pulses, we measured the amplitude of its response to the final pulse in each flicker. At the higher light intensity, wild-type cells' tracking ability tended to improve as the flicker frequency increased, and the ability to track the 0.5 Hz flicker increased significantly in the rod-functionless cells (Figure 7A *top*). At the lower intensity, wild-type cells tracked all three flicker frequencies more or less equally well, and tracking ability was improved in rod-functionless cells for all three frequencies (Figure 7A *bottom*).

The various genotypes' averaged flicker responses seemed to show different degrees of sustainability (Figure 6 first four *columns*). To quantify the sustainedness of the flicker responses, we computed ratios of the peak amplitude in the final-pulse response to the peak amplitude in the first-pulse response. The only significant change in flicker response sustainedness was seen when melanopsin was knocked out, making the cells' responses less sustained for the 0.5 Hz flicker at the higher intensity (Figure 7B *top left*).

**Figure 8:**
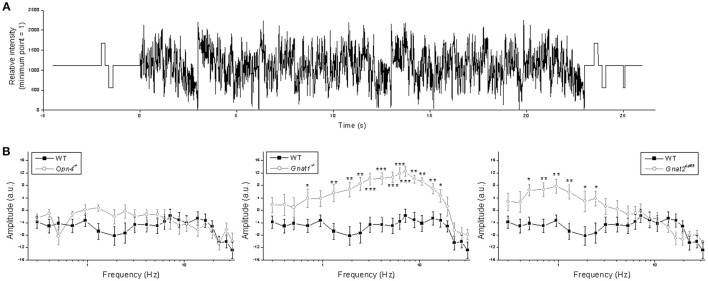
Responses to the sum-of-sinusoids stimulus. **(A)** The sum-of-sinusoids stimulus. **(B)** Frequency-specific response amplitude comparisons between the wild-type cells (*black squares*) and the other genotypes (*open circles*). *N* values were 15 cells for wild type, 10 cells for *Opn4*^−/−^, 9 cells for *Gnat1*^−/−^, and 9 cells for *Gnat2*^*cpfl*3^. ^*^p < 0.05, ^**^p < 0.01, ^***^p < 0.001.

The previous experiment tested only three frequencies and all pulses were delivered against a dark background, but the visual scene typically contains far more temporal frequencies and much of vision occurs in light-adapted conditions. Thus, in the next experiment we presented a sum-of-sinusoids stimulus with 21 frequencies summated around a 10.8 log photons cm^−2^ s^−1^ mean intensity (Figure 8A) to rapidly assay M4 cells' responses to a wider range of temporal frequencies under a somewhat more light-adapted state. The range of this stimulus (9.1 log−12.5 log photons cm^−2^ s^−1^) was chosen because it is just high enough to stimulate melanopsin to some degree (Figure 2 *rightmost column*) while probably low enough to avoid excessive bleaching of rods and cones. Fast Fourier transform was used to calculate each cell's response amplitude at each of the 21 frequencies found in the stimulus. As shown in Figure 8B, knocking out melanopsin had no significant effect at any of the frequencies, whereas eliminating rod or cone input significantly increased responses at 13 and 6 frequencies, respectively.

### Spatial visual behavior

**Figure 9:**
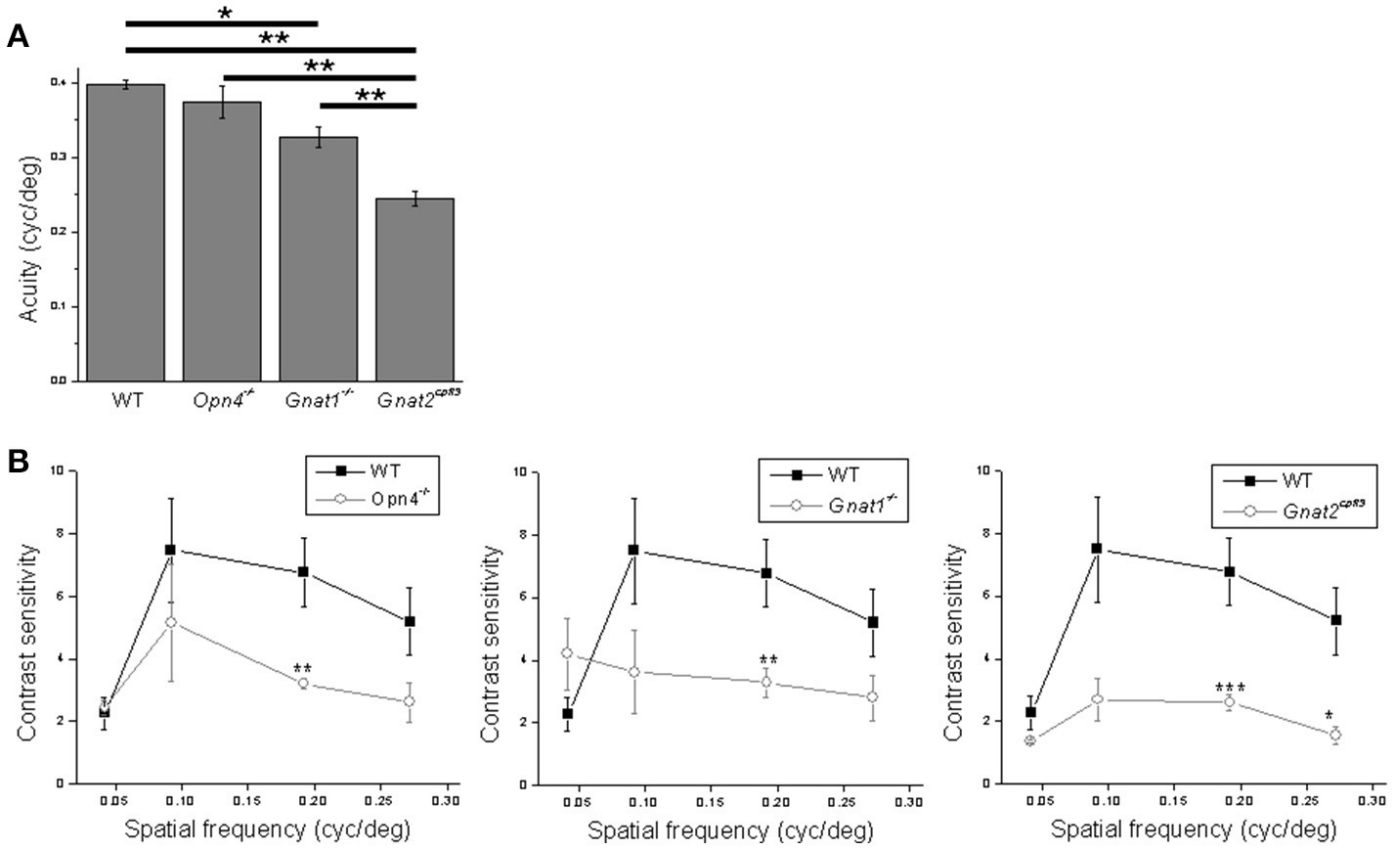
Analysis of the behavioral data. **(A)** Acuity was assessed using sine wave gratings with 100% contrast. **(B)** Contrast sensitivity was assessed using sine wave gratings with four different spatial frequencies. In both experiments all mice were male, 4–6 months old, and *n* = 5 mice per genotype for each testing condition. ^*^p < 0.05, ^**^p < 0.01, ^***^p < 0.001.

As mentioned in the Introduction, ipRGCs mediate not only non-image-forming behavioral responses but also pattern vision, as previously demonstrated using an optokinetic tracking assay (Schmidt et al., 2014). Thus, we concluded this study by examining how the optokinetic tracking behavior of mice was impacted by the elimination of melanopsin, rod, or cone function. In the first assay, we determined the various genotypes' acuity, i.e., the highest grating spatial frequency evoking a tracking response. Abolishing cone function reduced acuity most substantially, although disrupting rod function also caused a significant reduction. In contrast, knocking out melanopsin had no effect (Figure 9A).

In the second assay we determined the animals' contrast sensitivities for four different grating spatial frequencies. Contrast sensitivity was significantly reduced for the second highest spatial frequency in melanopsin-knockout, rod-functionless and cone-functionless mice, by comparable amounts. For the cone-functionless mice, contrast sensitivity was also significantly reduced at the highest spatial frequency (Figure 9B). Incidentally, the contrast sensitivities we measured were generally lower than those reported in Schmidt et al. (2014), which could be because we detected tracking responses by identifying longer (1–1.5 s) tracking episodes.

## Discussion

We have elucidated the functional necessity of rods, cones, and melanopsin by examining the physiologic and behavioral consequences of abolishing each of these photoreceptive components. One of the most significant effects was the dramatic reduction of the post-illumination excitation in M4 cells lacking rod input. We also saw clear deficits in visual acuity and contrast sensitivity. Surprisingly, many of the responses to temporally modulated stimuli were potentiated rather than reduced by the elimination of rod or cone input, indicating that an M4 cell's rod/cone-driven photoresponse may not be a simple summation of these inputs. In both the light-step and flicker experiments, relatively small effects were observed for final-to-peak amplitude ratios, possibly suggesting functional redundancy among rod input, cone input, and melanopsin. These findings are discussed in further detail below.

### Light-on response

M4 cells' responses to light-step onset were reduced at several intensities in the rod-functionless and cone-functionless cells but not the melanopsin-knockout cells, suggesting that only rod and cone inputs participate in this short-latency response, as expected from the sluggish onset of melanopsin-based photoresponses (Berson et al., 2002). However, the intensities at which light-on responses were reduced were somewhat lower in the rod-functionless cells than in the cone-functionless cells, consistent with rods being more photosensitive than cones (Fain and Dowling, 1973).

### Photoresponse sustainability

The observation that negative masking responses were shortened in melanopsin-knockout mice lent support to the notion that melanopsin is crucial for tonic ipRGC photoresponses (Mrosovsky and Hattar, 2003). Similarly, knocking out melanopsin rendered light responses in the suprachiasmatic nucleus (which receives ipRGC input) more transient (Mure et al., 2007), and light-evoked pupil constriction also became less tonic (Zhu et al., 2007; Keenan et al., 2016). But there were also reports of impressively long-lasting rod/cone-driven light responses in both the suprachiasmatic nucleus (van Oosterhout et al., 2012) and ipRGCs (Wong, 2012), so the relative importance of melanopsin vs. rod/cone signaling in photoresponse sustainability remained unresolved. Here we found that only rod and cone inputs contribute significantly to the sustainedness of M4 cells' responses to 10-s light steps at certain intensities. For the flickering stimuli, however, eliminating rod or cone activity does not impact the sustainedness of responses to any of the flickers tested, while knocking out melanopsin makes responses to one of the three higher-intensity flickers less sustained.

Taken together, the present observations suggest that some of the seemingly contradictory results in previous papers regarding response sustainability could be partly due to differences in photostimulation conditions such as intensities, durations, and temporal characteristics. Functional differences among the ipRGC types and post-retinal processing (Keenan et al., 2016) could also add to the discrepancies between ipRGC recordings and behavioral data.

### Post-illumination persistence

We found that the rod input to M4 cells is responsible for the majority of their poststimulus depolarization; in fact, melanopsin does not seem to contribute at all. Early recordings showed that rod/cone-driven ipRGC responses to light steps had rapid offset (Dacey et al., 2005; Perez-Leon et al., 2006; Wong et al., 2007; Schmidt and Kofuji, 2010), leading to the hypothesis that the poststimulus persistence of the ipRGC photoresponse is mostly if not entirely due to melanopsin. Indeed, the post-illumination sustained pupil responses in primates were reported to have melanopsin-like wavelength sensitivity (Gamlin et al., 2007), forming the basis for the now-widespread use of this persistent response to assess melanopsin function in humans (Kankipati et al., 2010; Park et al., 2011). However, the subjects in Gamlin et al. (2007) were partially light-adapted, potentially limiting rods' contribution to the pupil responses. Considering our present data, investigators using the post-illumination pupil response to measure melanopsin function should minimize rod contribution to the response, either by using a rod-saturating background or by limiting the duration of pre-testing dark adaptation.

Contrary to our results, an earlier paper reported that knocking out melanopsin dramatically reduced the poststimulus persistence of M4 cells' light-step responses (Schmidt et al., 2014). Both that study and ours used retinas that had been isolated from the RPE so that bleached rhodopsin could not be replenished through the retinoid cycle (Wang and Kefalov, 2011), but our light steps were one third in duration and we tested fewer stimuli at high intensities (11.5 log−15.5 log photons cm^−2^ s^−1^), so rods were likely less desensitized in our recordings. By contrast, because melanopsin has a much higher threshold than rhodopsin and is comparatively resistant to photobleaching (Sexton et al., 2012; Emanuel and Do, 2015; Zhao et al., 2016), it was probably well preserved in both studies. Consequently, Schmidt et al. probably overestimated the importance of melanopsin relative to the rod input.

### Tracking of temporally modulated stimuli

Using melanopsin alone, ipRGCs can track flickering light up to only ~0.2 Hz (Walch et al., 2015). In the present study, all the frequencies tested in the flicker and sum-of-sinusoids experiments were above 0.2 Hz and thus should not be trackable by melanopsin, and indeed knocking out melanopsin did not significantly affect any of the frequencies examined.

For both flickering and sum-of-sinusoids stimuli, tracking was enhanced at many frequencies in M4 cells lacking rod input, which is consistent with a reduction in the poststimulus response persistence, thereby allowing the cell to recover from the previous light pulse more rapidly. In the sum-of-sinusoids experiment, silencing rods affected M4 cells' responses at up to 16.7 Hz, which may appear to contradict classic studies reporting the temporal acuity of rod-mediated vision to be at most 15 Hz (Hecht and Shlaer, 1936), but more recent studies showed that rods can track stimuli at 28 Hz or even higher frequencies (Conner and MacLeod, 1977; Nusinowitz et al., 2007).

For M4 cells lacking cone input, the flicker and sum-of-sinusoids experiments yielded seemingly contradictory results: responses to the flickers were not significantly affected at 0.5, 2, or 5 Hz, but responses to the sum-of-sinusoids increased at all frequencies between 0.5 and 2.45 Hz. This can be explained by cones influencing rods via gap-junction coupling (Raviola and Gilula, 1973). Unlike the flickers which were delivered in darkness, the sum-of-sinusoids stimulus was presented against a background light, and in wild-type retina this background hyperpolarizes rods and cones, thereby compressing their responses to the sinusoids. Rod responses to the sinusoids are presumably further compressed as cones transmit their background-evoked hyperpolarization to rods via gap junctions. Although cones are known to adapt rapidly to prolonged illumination, they maintain a steady-state hyperpolarization about half the size of the peak hyperpolarization (Normann and Perlman, 1979; Burkhardt, 1994), enabling them to continuously suppress the coupled rods. But *Gnat2*^*cpfl*3^ cones lack the G-protein that mediates phototransduction (Chang et al., 2006), so they are constitutively depolarized and do not add to the background-induced compression in the coupled rods, ultimately allowing larger rod-driven photoresponses in downstream ipRGCs. Consistent with this explanation, cone-driven electroretinograms measured in the presence of a background light are substantially larger in *Gnat1*^−/−^ mice than in wild-type mice, probably because rods in the *Gnat1*^−/−^ mice remain depolarized and thus no longer suppress cones through gap junctions (Cameron and Lucas, 2009). The lack of wild-type *vs. Gnat2*^*cpfl*3^ differences outside the 0.5–2.45 Hz range (Figure 8B *right*) could reflect the bandpass properties of rod-cone coupling.

### Image-forming vision

Using an optokinetic tracking assay identical to ours, a previous study detected a role for melanopsin in contrast sensitivity (Schmidt et al., 2014). Our results showed that at least for some spatial frequencies, melanopsin's role in contrast sensitivity may be as substantial as rods' and cones'. The promotion of contrast sensitivity by melanopsin could theoretically be mediated by not only ipRGCs' projections to image-forming visual centers, but also their intraretinal signaling to amacrine cells which subsequently regulate image-forming retinal circuits (Hankins and Lucas, 2002; Allen et al., 2014). Two intraretinal ipRGC signaling pathways have been identified: glutamatergic transmission from M1 cells to a subset of dopaminergic amacrine cells (Zhang et al., 2008; Prigge et al., 2016), and gap junctional transmission from multiple types of ipRGCs to some non-dopaminergic amacrine cells (Muller et al., 2010; Reifler et al., 2015a; Chervenak et al., 2016). Although Schmidt and colleagues ruled out the involvement of M1 cells and hence the glutamatergic pathway in the modulation of contrast sensitivity (Schmidt et al., 2014), the involvement of the gap junctional pathway remains possible.

On the other hand, we found spatial acuity to be determined by rods and cones alone, consistent with the absence of optokinetic tracking in rod/cone-functionless, melanopsin-only mice (Ecker et al., 2010). For acuity, cones were found to be more important than rods as silencing cones reduced acuity by a greater amount than silencing rods. This is expected since the stimulus intensity at the cornea, >13 log photons cm^−2^ s^−1^, was well within the photopic range (Dacey et al., 2005). Nonetheless, rods did contribute significantly to acuity, which may seem surprising given the photopic conditions. A previous study found that mice possessing functional rods but lacking functional cones and melanopsin continued to exhibit optokinetic reflexes in photopic conditions as long as the pupils were allowed to constrict (Cahill and Nathans, 2008). More recent work has further shown that even though daylight conditions initially saturate rods, these cells gradually escape saturation and regain contrast sensitivity (Tikidji-Hamburyan et al., 2017).

In both the flicker and sum-of-sinusoids experiments, responses at many frequencies were increased rather than decreased in mice lacking rod or cone function, but these mice never showed enhanced acuity or contrast sensitivity in the optokinetic assay—the apparent contrast sensitivity increase at the lowest spatial frequency in *Gnat1*^−/−^ mice (Figure 9B *middle*) was statistically insignificant. But a direct comparison is impossible because optokinetic tracking is driven by multiple types of ganglion cells (Berson, 2008), and testing conditions (e.g., adaptational states, and stimulus durations, waveforms and intensities) also differed between the single-cell and behavioral experiments.

### Additional functions of melanopsin?

At least two more roles of melanopsin have been suggested in the literature. An early study observed reduced pupil constriction at high irradiances in melanopsin-knockout mice (Lucas et al., 2003). But in our light-step experiment, we did not detect any reduction in the peak response amplitude or final-to-peak amplitude ratio when melanopsin was knocked out in M4 cells. While Schmidt and colleagues did detect reduced light-evoked spiking in melanopsin-knockout M4 cells at one irradiance, responses at higher irradiances were wild-type-like (Schmidt et al., 2014). This apparent discrepancy between single-cell recordings and a behavioral output could reflect differences between M4 and M1, the main ipRGC type driving pupillary constriction (Ecker et al., 2010).

Schmidt and colleagues showed that while wild-type M4 cells' spike rates could follow the intensity changes in a stimulus that first became brighter over 6 min and then dimmer over 6 min, melanopsin-knockout M4 cells could only signal the upward ramp and stopped spiking shortly after the stimulus began to dim, suggesting melanopsin is needed to faithfully encode irradiance (Schmidt et al., 2014). But as discussed above in the context of post-illumination persistence, a caveat is that these recordings were made under conditions where rhodopsin was irreversibly bleached by light. Thus, by the time the downward ramp started, most rhodopsin molecules could have already been bleached by the preceding 6 min of illumination, leaving melanopsin as the only photopigment capable of signaling the intensity decrement. It remains to be tested how well melanopsin-knockout M4 cells can signal intensity reduction under conditions permitting rhodopsin regeneration.

### Limitations of this study

Our central strategy was to assess whether eliminating each photoreceptive system would significantly alter certain parameters of the ipRGC photoresponse or spatial visual behavior. Because the disruption of photosensitivity was unconditional, it could have induced developmental alterations of retinal circuits (Rao et al., 2013) and perhaps even mild degeneration in the *Gnat2*^*cpfl*3^ retinas (Chang et al., 2006). Despite this potential caveat, our results are largely consistent with known properties of the various photoreceptors, as discussed above. We had tried to use a small-molecule antagonist of melanopsin phototransduction, AA92593, to acutely block melanopsin-based light responses (Jones et al., 2013), but in our hands this drug failed to significantly reduce these responses. Lucas et al. have studied the roles of melanopsin and cones by using melanopsin- and cone-selective light stimuli (Lall et al., 2010; Allen et al., 2014), thereby avoiding any developmental complications in knockout mice, but this approach cannot be used to achieve our present goal, i.e., eliminating melanopsin, cone or rod activity to evaluate the functional necessity of each photoreceptor class.

Another limitation is that all light-step and flicker responses were measured under scotopic conditions. Though a steady background was embedded in the sum-of-sinusoids stimulus, this background was in the mesopic range and so the M4 cells likely remained fairly well dark-adapted. We performed all whole-cell recordings under scotopic/mesopic conditions and limited stimulus durations to 10 s (for the light steps), 20 s (for the flickers), and 23 s (for the sum-of-sinusoids stimulus) to minimize irreversible bleaching of rod and cone photopigments, as the isolated retinas used in this study did not have access to the RPE's retinoid cycle.

Finally, we examined only M4 cells and the findings may not be applicable to all the other ipRGC types. For instance, melanopsin could conceivably contribute more prominently to the light responses of M1–M3 ipRGCs, which express this photopigment at higher levels than M4 cells (Berson et al., 2010; Estevez et al., 2012). Moreover, M2 cells have been found to exhibit larger cone-driven light responses than M1 cells (Schmidt and Kofuji, 2010), suggesting that cone signaling may differentially impact the various ipRGC types.

## Summary

We have found that, with one exception, melanopsin is largely dispensable for the light response of M4 ipRGCs under our testing conditions, even though our stimuli included intensities above the melanopsin activation threshold. Rod and cone inputs contribute to several aspects of M4 cells' photoresponse, with the rod input being solely responsible for the post-illumination persistence of the response. At the behavioral level, all three photoreceptor classes are important for contrast sensitivity, whereas visual acuity is established only by rods and cones. Since we examined just the M4 type, future studies will be needed to ascertain whether our single-cell results are applicable to the other types of ipRGCs.

## Author contributions

KW designed research. All authors performed research. KH, XZ, and KW analyzed data. KW wrote the paper.

### Conflict of interest statement

The authors declare that the research was conducted in the absence of any commercial or financial relationships that could be construed as a potential conflict of interest. The reviewer ES and handling Editor declared their shared affiliation.
